# Patients With Pancreatic Cancer With Synchronous Liver Oligometastasis Show a Significant Long‐Term Survival Benefit From Resection Under Specific Conditions: A Multicenter National Cohort Study

**DOI:** 10.1002/jhbp.12181

**Published:** 2025-07-13

**Authors:** Kohei Nakata, Yoshihiro Miyasaka, Takeaki Ishizawa, Masayuki Ohtsuka, Masamichi Mizuma, Sohei Satoi, Masaaki Hidaka, Shuji Suzuki, Hiroshi Kurahara, Chie Kitami, Satoshi Hirano, Dongha Lee, Saiho Ko, Munenori Tahara, Isaku Yoshioka, Kenjiro Date, Kazuyuki Nagai, Goro Honda, Shugo Mizuno, Kenichi Hakamada, Yasuro Futagawa, Shigeru Marubashi, Hiroshi Yoshida, Akihiko Horiguchi, Yasuo Hosouchi, Masafumi Imamura, Naoto Gotohda, Hiroaki Nagano, Masaji Tani, Takeshi Sudo, Teijiro Hirashita, Junichi Arita, Katsutoshi Murase, Ken Fukumitsu, Toshiki Rikiyama, Teruyuki Usuba, Toshiya Abe, Masafumi Nakamura, Itaru Endo

**Affiliations:** ^1^ Department of Surgery and Oncology, Graduate School of Medical Sciences Kyushu University Fukuoka Japan; ^2^ Department of Surgery Fukuoka University Chikushi Hospital Chikushino Japan; ^3^ Department of Hepatobiliary‐Pancreatic Surgery, Graduate School of Medicine Osaka Metropolitan University Osaka Japan; ^4^ Department of General Surgery Chiba University Graduate School of Medicine Chiba Japan; ^5^ Department of Surgery Tohoku University Graduate School of Medicine Sendai Japan; ^6^ Department of Pancreatobiliary Surgery Kansai Medical University Osaka Japan; ^7^ Department of Digestive and General Surgery Shimane University Faculty of Medicine Izumo Shimane Japan; ^8^ Department of Gastroenterological Surgery, Ibaraki Medical Center Tokyo Medical University Ibaraki Japan; ^9^ Department of Digestive Surgery, Graduate School of Medical and Dental Sciences Kagoshima University Kagoshima Japan; ^10^ Department of Surgery, Nagaoka Chuo General Hospital Nagaoka Japan; ^11^ Department of Gastroenterological Surgery II Hokkaido University Faculty of Medicine Sapporo Hokkaido Japan; ^12^ Department of Surgery Kindai University Faculty of Medicine Osakasayama Japan; ^13^ Department of Surgery, Nara Prefecture General Medical Center Nara Japan; ^14^ Department of Surgery, Sapporo‐Kosei General Hospital Sapporo Japan; ^15^ Department of Surgery and Science, Faculty of Medicine, Academic Assembly University of Toyama Toyama Japan; ^16^ Department of Surgery, Kitakyushu Municipal Medical Center Kitakyushu Japan; ^17^ Department of Surgery, Graduate School of Medicine Kyoto University Kyoto Japan; ^18^ Department of Surgery Institute of Gastroenterology, Tokyo Women's Medical University Tokyo Japan; ^19^ Department of Hepatobiliary Pancreatic & Transplant Surgery Mie University Tsu Mie Japan; ^20^ Department of Gastroenterological Surgery Hirosaki University Graduate School of Medicine Hirosaki Japan; ^21^ Department of Surgery Jikei University Daisan Hospital Tokyo Japan; ^22^ Department of Hepato‐Biliary‐Pancreatic and Transplant Surgery Fukushima Medical University Fukushima Japan; ^23^ Department of Surgery, Iwaki City Medical Center Iwaki Japan; ^24^ Department of Gastroenterological Surgery Fujita Health University Bantane Hospital Nagoya Japan; ^25^ Department of Surgery, Gunma Prefecture Saiseikai Maebashi Hospital Maebashi Japan; ^26^ Department of Surgery, Surgical Oncology and Science Sapporo Medical University School of Medicine Sapporo Hokkaido Japan; ^27^ Department of Hepatobiliary and Pancreatic Surgery, National Cancer Center Hospital East Kashiwa Japan; ^28^ Department of Gastroenterological, Breast and Endocrine Surgery Yamaguchi University Graduate School of Medicine Yamaguchi Japan; ^29^ Department of Surgery Shiga University of Medical Science Otsu Shiga Japan; ^30^ Department of Surgery NHO Kure Medical Center Chugoku Cancer Center Hiroshima Japan; ^31^ Department of Gastroenterological and Pediatric Surgery Oita University Faculty of Medicine Oita Japan; ^32^ Department of Gastroenterological Surgery Akita University Graduate School of Medicine Akita Japan; ^33^ Department of Gastroenterological Surgery, Graduate School of Medicine Gifu University Gifu Japan; ^34^ Department of Surgery, Kyoto Katsura Hospital Kyoto Japan; ^35^ Department of Surgery, Saitama Medical Center Jichi Medical University Saitama Japan; ^36^ Department of Surgery, Katsushika Medical Center The Jikei University School of Medicine Minato‐ku Tokyo Japan; ^37^ Department of Gastroenterological Surgery, Graduate School of Medicine Yokohama City University Yokohama Japan; ^38^ Japan Society of Hepato‐Biliary‐Pancreatic Surgery Osaka Japan

**Keywords:** oligometastasis, pancreatic cancer, preoperative chemotherapy

## Abstract

**Background:**

In this study, we investigated the criteria that predict the long‐term survival benefits after surgical resection in patients with pancreatic cancer accompanied by liver oligometastasis.

**Methods:**

In total, 60 patients from 34 high‐volume Japanese centers who underwent surgical resection for liver oligometastasis between 2005 and 2020 were included. Univariate and multivariate methods of survival analyses were performed. All patients were followed up for at least 36 months.

**Results:**

Overall survival (OS) was significantly longer in the preoperative chemotherapy group than in the up‐front surgery group (37.4 vs. 20.4 months, *p* = 0.001). In the operation with preoperative chemotherapy group, a complete response was observed in eight patients (28.6%). The 1‐, 3‐, and 5‐year OS rates were 92.9%, 50.0%, and 35.7%, respectively. The multivariate analysis showed that low CA19‐9 (< 100 U/mL; HR: 0.25; 95% CI: 0.06–0.96; *p* = 0.043), low CEA (< 5 U/mL; HR: 0.14; 95% CI: 0.04–0.48; *p* = 0.002), and resectable (R) or borderline resectable pancreatic cancer invading the portal vein (BR‐PV) status (HR: 0.19; 95% CI: 0.07–0.51; *p* < 0.001) were positive prognostic factors. The median OS of the patients who met all three criteria was 106.6 months.

**Conclusion:**

Preoperative chemotherapy is essential for the treatment of liver oligometastases. Despite the high recurrence rates, patients who met the specific criteria have a favorable prognosis with liver resection.

## 1 Introduction

Pancreatic cancer has a much lower 5‐year survival rate (13%) than that of other solid cancers. Although surgical resection with adjuvant chemotherapy is the only curative treatment for pancreatic cancer, 50% of patients have metastatic lesions [1, 2]. The most common site of synchronous metastasis is the liver (77%–88.7%), followed by lungs (10.1%) and bones (2.0%) [3, 4]. The presence of liver metastasis at the time of pancreatic cancer diagnosis was reported to be 35.6%–40% [3, 5]. These cases have been considered contraindications for surgical resection [6]; however, recent developments in multidisciplinary treatment have led to successful resections after chemotherapy. Given that the reported prognosis of patients with liver metastasis who undergo surgery following preoperative chemotherapy ranges from 36.7 to 56 months [7, 8, 9], surgical treatment can be justified for these patients. However, the precise preoperative clinical factors that predict the long‐term prognosis are not well known.

Recently, there has been increasing attention on oligometastasis, a state characterized by a limited metastatic spread that represents the intermediate stage between localized disease and widespread metastasis [10, 11]. This concept has significant implications for cancer treatment because patients with oligometastases may benefit from more aggressive local therapies including surgical treatment [9, 10, 12]. However, surgical indications for patients with liver oligometastases have varied across institutions and over time, making it challenging to establish a standardized criterion, and there are limited reports on the treatment of liver oligometastatic pancreatic cancer with surgical procedures because systemic chemotherapy is usually selected for patients with liver metastasis based on the current guidelines [6]. Furthermore, there have been no reports focusing only on a series of resected liver oligometastases of pancreatic cancer, nor have any reports specified the criteria that must be met for patients to achieve a long‐term prognosis after surgery.

In this study, we collected the clinicopathological data of patients with liver oligometastases of pancreatic cancer from highly experienced hospitals in Japan and investigated the conditions under which patients may benefit from longer survival to identify the optimal surgical indication for such patients.

## 2 Methods

### 2.1 Study Design and Data Collection

This multicenter study was conducted as a part of a project of the Japanese Society of Hepato‐Biliary‐Pancreatic Surgery (JSHBPS). In this study, data from 96 patients from 34 Japanese high‐volume centers who underwent simultaneous surgical resection of liver oligometastasis between 2005 and 2020 were collected. Surgical indications for patients with liver oligometastases have varied across institutions and over time. Oligometastasis was defined as the presence of three or fewer liver metastases [7, 8]. Of the 96 cases collected, 36 cases with metachronous oligometastasis, peritoneal dissemination, or non‐oligometastasis were excluded from this study; therefore, a total of 60 cases of liver oligometastasis were eligible for the study. The patients were divided into two groups: the upfront surgery group (*n* = 32) where patients did not receive preoperative chemotherapy, and the preoperative chemotherapy group (*n* = 28), where patients received chemotherapy or chemoradiotherapy before surgical treatment. Four patients who initially underwent resection of the liver metastasis for diagnosis followed by chemotherapy before primary tumor resection were included in the preoperative chemotherapy group. The database included the following preoperative variables: age, sex, body mass index (BMI), American Society of Anesthesiologists (ASA) class, presence of diabetes before surgery, number of liver metastases, operation procedures, nutritional status, tumor location, tumor size, tumor markers (carcinoembryonic antigen (CEA) and CA19‐9), and resectability before operation.

### 2.2 Resectablity of Pancreatic Cancer

The resectability of the primary tumor was defined based on the National Comprehensive Cancer Network Guidelines. The pancreatic carcinoma was classified by the Japan Pancreatic Society as resectable (R), borderline resectable (BR), or unresectable (UR). The BR category was further divided into two types: (i) BR‐A (arterial invasion): tumor contact or invasion of the superior mesenteric artery (SMA) and/or celiac artery (CA) of < 180° without stenosis or deformity, or tumor contact or invasion of the common hepatic artery (CHA) without involvement of the proper hepatic artery (PHA) and/or CA. (ii) BR‐PV (portal vein involvement): no findings of contact or invasion of the SMA, CA, or CHA, but tumor contact or invasion of the SMV/PV of 180° or more, or occlusion of the SMV/PV not exceeding the inferior border of the duodenum [6, 13].

The endpoint of this study was to identify the factors associated with a long survival time in patients with simultaneous liver oligometastasis of pancreatic cancer. This study was approved by the Ethical Board of Kyushu University (approval number 22058) and the Institutional Review Board of each participating institution. Case report forms (CRFs) were created and sent to participating institutions and collected and analyzed at the Department of Surgery and Oncology, Kyushu University. Overall survival (OS) was defined as the time from the initial treatment to death from any cause. Recurrence‐free survival (RFS) was defined as the time from surgery to tumor recurrence.

### 2.3 Statistical Analysis

The *χ* ^2^ test was used to compare categorical variables. Continuous variables were expressed as median values and compared using the Student's *t*‐test or Wilcoxon test. A *p‐*value of < 0.05 was considered statistically significant. Survival outcomes were calculated using the Kaplan–Meier method and compared using the log‐rank test. Only the statistically significant variables in the univariate analysis were included in the multivariate analysis, which was performed using a Cox proportional hazards regression model. Statistical analyses were performed and graphical representations were created using JMP 16 software (SAS Institute, Cary, NC, USA).

## 3 Results

### 3.1 Patient Background and Factors Associated With Survival in the Whole Cohort

Of the 96 patients, 60 with liver oligometastases were eligible for inclusion in the study; their clinical characteristics are summarized in Table 1. The number of oligometastases was one in most of the patients (80.0%), followed by two in nine patients (15.0%), and three in three patients (5.0%). Thirty‐two patients underwent upfront surgery between 2005 and 2019, while 28 patients received preoperative chemotherapy between 2010 and 2020. The median size of the primary tumor at diagnosis was 30 mm (range: 15–160 mm). Regarding the resectability, 44 patients were categorized as R, 12 as BR (BR‐PV in five and BR‐A in seven), and four as locally advanced (LA). A resectability status higher than BR‐A was more frequent in the surgery after preoperative chemotherapy group than in the up‐front surgery group (*p* = 0.001). However, tumor markers, tumor size, tumor location, and nutritional status did not differ between the two groups (Table 1).

**TABLE 1:** Patient characterictics.

Characteristics	All (*n* = 60)	Up‐front surgery (*n* = 32)	Preoperative chemotherapy (*n* = 28)	*p*
Age, median, years	67 (36–89)	68 (41–89)	66 (36–81)	0.680
≥ 70 years	22 (37.3%)	12 (37.5%)	10 (37.0%)	0.970
Gender (Male)	31 (51.7%)	11 (34.4%)	20 (71.4%)	0.004
BMI, median, kg/m^2^	21.6 (15.1–29.6)	21.3 (15.1–29.6)	23.1 (16.2–28.7)	0.300
ASA‐PS (I/II/III/IV/V)	33/26/1/0/0	20/12/0/0/0	13/14/1/0/0	0.250
Diabetes	19 (31.7%)	9 (28.1%)	10 (35.7%)	0.529
Number of liver metastasis (1/2/3)	48/9/3	26/4/2	22/5/1	0.772
Resectability of primary tumor (R/BR‐PV/BR‐A/UR)	44/5/7/4	28/3/1/0	16/2/6/4	0.005
R, BR‐PV/BR‐A, UR	49/11	31/1	18/10	0.001
PNI	46.2 (35.3–55.6)	46.3 (35.3–54.0)	45.7 (38.9–55.6)	0.580
NLR	2.8 (0.9–11.1)	2.8 (1.4–11.1)	2.6 (0.9–7.8)	0.679
mGPS (0/1/2)	48/11/1	24/7/1	24/4/0	0.378
Tumor location (head/body or tail)	29/31	19/13	10/18	0.066
Size of primary tumor (mm)	30 (15–160)	30 (15–50)	30 (16–160)	0.330
CEA at diagnosis	3.75 (0.6–110.8)	4.3 (1.1–110.8)	3.5 (0.6–30)	0.182
< 2.5	18	9	9	
2.5 ≤ < 5.0	19	8	11	
5.0 ≤	23	15	8	
CA19‐9 at diagnosis	154.5 (0–18 091)	219.65 (0.1–18 091)	93 (0–4704)	0.051
< 37.0	13	5	8	
37.0 ≤ < 100	12	6	6	
100 ≤	35	21	14	
*Preoperative chemotherapy*				
Duration (M)			5.5 (1.5–15)	
Gem+nabPTX			13	
Gem+S‐1			11	
mFFX			3	
Gem			1	
Size of primary tumor after treatment (mm)			22 (0–104)	—
CEA after preoperative treatment			3.35 (0.7–42.3)	
CA19‐9 after preoperative treatment			22.7 (0.6–2242)	
Operative procedure of liver resection (PR/segementectomy)	57/3	32/0	25/3	0.058
Operative procedure (DP/PD/TP)	30/29/1	12/20/0	18/9/1	0.037
pT (T1/2/3/4)	7/14/34/4	0/7/23/2	7/7/11/2	0.004
pN (0/1/2)	25/25/10	10/13/9	15/12/1	0.017
pR0	50 (90.6%)	29 (90.6%)	21 (75.0%)	0.106
CEA after operation	2.5 (0.6–658.4)	2.5 (0.9–18)	2.6 (0.6–658.4)	0.581
CA19‐9 after operation	30 (0.1–8322.6)	49 (0.1–2947)	19.4 (0.6–8322.6)	0.197

Abbreviations: ASA‐PS, American Society of Anesthesiologists physical status; BMI, body mass index; DP, distal pancreatectomy; mGPS, modified Glasgow Prognostic Score; NLR, neutrophil‐to‐lymphocyte ratio; PD, pancreatoduodenectomy; PNI, prognostic nutritional index; PR, partial resection; TP, total pancreatectomy.

**FIGURE 1:**
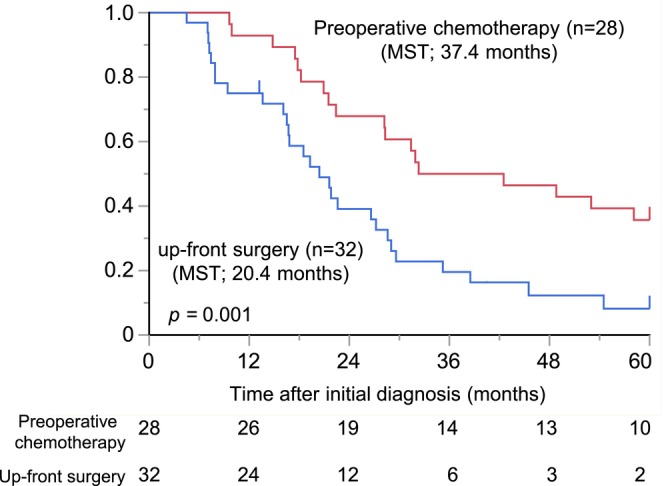
Overall survival in patients with pancreatic ductal adenocarcinoma (PDAC) who underwent preoperative chemotherapy and up‐front surgery. Patients alive at the last follow‐up are censored.

To assess the necessity and effectiveness of preoperative chemotherapy in patients with liver oligometastatic pancreatic cancer, we initially compared the difference in prognosis between the up‐front surgery group and preoperative chemotherapy group. The OS was significantly longer in the preoperative chemotherapy group than in the up‐front surgery group (median survival time (MST): 37.4 vs. 20.4 months, *p* = 0.001, Figure 1a). The recurrence rate was higher in the up‐front surgery group (30/32, 93.7%) than in the preoperative chemotherapy group (20/28, 71.4%) (*p* = 0.018). The results of the Cox proportional hazards regression model for prognostic factors of OS are shown in Table 2. Preoperative chemotherapy was the only independent prognostic factor in multivariate analyses (hazard ratio (HR): 0.50; 95% confidence interval (CI): 0.27–0.90; *p* = 0.020) These findings suggest that preoperative chemotherapy is essential for patients with liver oligometastases.

**TABLE 2:** Urivariate and multivariate analysis of prognostic factors for OS in patients with pancreatic cancer.

Factors	Description	Patients *n*	Urivariate analysis, hazard ratio (95% CI)	*p*	Multivariate analysis, hazard ratio (95% CI)	*p*
Age, median, years	≧ 70/< 70 years		1.69 (0.94–3.01)	0.075		
Gender (male)	Male/female		0.63 (0.36–1.11)	0.109		
BMI, median, kg/m^2^	≧ 22/< 22		1.09 (0.62–1.90)	0.771		
ASA‐PS (I/II/III/IV/V)	1/2, 3		1.12 (0.64–1.96)	0.682		
Diabetes	Yes/no		0.96 (0.53–1.75)	0.905		
Jaundice	Yes/no		1.30 (0.73–2.32)	0.380		
Number of liver metastasis (1/2/3)	2,3/1		1.13 (0.57–2.27)	0.725		
Resectability of primary tumor (R/BR‐PV/BR‐A/UR)	R, BR‐PV/BR‐A, LA		0.67 (0.34–1.32)	0.249		
PNI	< 45/≧ 45		1.37 (0.76–2.48)	0.299		
NLR	< 5/5 ≤		0.63 (0.24–1.61)	0.331		
mGPS (0/1/2)	0/1, 2		1.00 (0.50–2.00)	0.996		
Tumor locarion (head/body or tail)			1.29 (0.74–2.24)	0.364		
CEA at diagnosis						
	< 5.0/5.0 ≤ng/ml		0.60 (0.34–1.07)	0.086		
CA19‐9 at diagnosis						
	< 100/100 ≤ U/mL		0.50 (0.28–0.90)	0.020		
Operation	PD, TP/DP		1.38 (0.80–2.41)	0.248		
Preoperative chemotherapy	Yes/no		0.43 (0.25–0.77)	0.004*	0.50 (0.27–0.90)	0.020*

Abbreviations: ASA‐PS, American Society of Anesthesiologists physical status; BMI, body mass index; DP, distal pancreatectomy; mGPS, modified Glasgow Prognostic Score; NLR, neutrophil‐to‐lymphocyte ratio; PD, pancreatoduodenectomy; PNI, prognostic nutritional index; TP, total pancreatectomy.

* *p* values are statistically significant.

### 3.2 Effectiveness of Preoperative Chemotherapy for Patients With Liver Oligometastasis

The median duration of preoperative chemotherapy was 5.5 months (range: 1.5–15 months). The chemotherapy regimens were GnP (gemcitabine, nab‐paclitaxel) in 13 patients, GS (gemcitabine, tegafur‐gimeracil‐oteracil potassium) in 11 patients, modified FOLFIRINOX (mFFX) in three patients, and gemcitabine (GEM) in one patient. The effect of chemotherapy on the primary tumor was complete response (CR) in one case, partial response (PR) in 12 cases, stable disease (SD) in 14 cases, and progressive disease (PD) in one case. Regarding liver oligometastasis, all except for two cases (92.9%) showed a decrease in the size of the metastases. Notably, CR for liver metastasis was observed in eight cases (28.6%), with no tumor cells in the pathological diagnosis. Among these patients, only one achieved CR for the primary tumor. The chemotherapy regimens in patients with CR for liver oligometastasis were GnP in five cases, GS in two cases, and mFFX in one case. The duration of chemotherapy was significantly longer in patients with CR for liver oligometastasis (12 months; 3–15 months) than in those without non‐CR for liver oligometastasis (3.1 months; 1.5–13.5 months) (*p* = 0.011).

### 3.3 Factors Associated With Survival in Patients With Preoperative Chemotherapy

**FIGURE 2:**
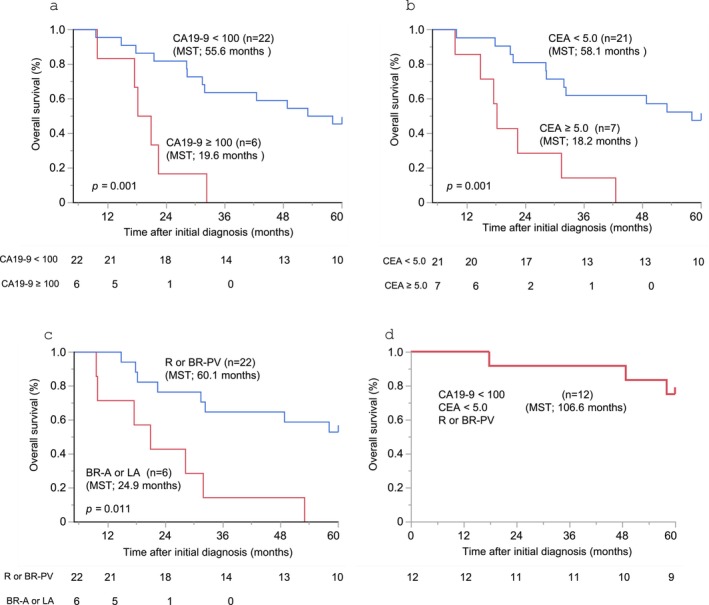
Overall survival in patients with liver oligometastatis with (a) low CA19‐9 and high CA19‐9, (b) low CEA and high CEA, and (c) R or BR‐PV and BR‐A or UR. (d) Overall survival in patients with pancreatic ductal adenocarcinoma (PDAC) who met all the criteria.

Following operation, adjuvant chemotherapy was administered to 24 patients (85.7%). The regimens were S‐1 in 15 patients, GEM in five, GnP in three, and GS in one. The median OS was 37.4 months. The 1‐, 3‐, and 5‐year OS rates were 92.9%, 50.0%, and 35.7%, respectively. The univariate analysis showed that low CA19‐9 levels (< 100 U/mL), low CEA levels (< 5 U/mL), and resection status with R‐ or BR‐PV after chemotherapy were positive prognostic factors (Table 3). The median OS after initial diagnosis was 55.6, 58.1, and 60.1 months, respectively (Figure 2a–c). The multivariate analysis for OS also showed that low CA19‐9 (< 100 U/mL) (HR: 0.25; 95% CI: 0.06–0.96; *p* = 0.043), low CEA (< 5 U/mL) (HR: 0.14; 95% CI: 0.04–0.48; *p* = 0.002) and R or BR‐PV resection status (HR: 0.19; 95% CI: 0.07–0.51; *p* < 0.001) were positive prognostic factors (Table 3). There were 12 patients who met all three criteria; the median OS in these patients was 106.6 months, and the 1‐, 3‐, and 5‐year OS rates were 100%, 91.7%, and 75.0%, respectively (Figure 2d).

**TABLE 3:** Urivariate and multivariate analysis of prognostic factors for OS in patients with pancreatic cancer.

Factors	Description	Patients, *n*	Urivariate analysis, hazard ratio (95% CI)	*p*	Multivariate analysis, hazard ratio (95% CI)	*p*
Age, median, years	≧ 70/< 70 years		2.18 (0.90–5.28)	0.085		
Gender (male)	Male/female		0.64 (0.26–1.60)	0.338		
BMI, median, kg/m^2^	≧ 22/< 22		1.77 (0.67–4.66)	0.246		
ASA‐PS (I/II/III/IV/V)	1/2, 3		0.57 (0.23–1.41)	0.222		
Diabetes	Yes/no		1.57 (0.65–3.82)	0.317		
Number of liver metastasis (1/2/3)	2,3/1		1.47 (0.53–4.12)	0.458		
Preoperative chemotherapy	< 3 M/3 M ≤		1.32 (0.50–3.47)	0.571		
	< 6 M/6 M ≤		1.45 (0.61–3.46)	0.401		
Effectiveness of chemotherapy	PD, SD/PR, CR*		1.91 (0.77–4.70)	0.161		
mCR	nonCR/CR		1.20 (0.47–3.08)	0.706		
Resectability of primary tumor (R/BR‐PV/BR‐A/UR)	R, BR‐PV/BR‐A, LA		0.40 (0.18–0.90)	0.026*	0.19 (0.07–0.51)	< 0.001*
PNI	< 45/45 ≤		2.03 (0.84–4.93)	0.114		
NLR	< 5/5 ≤		0.46 (0.13–1.62)	0.226		
mGPS (0/1/2)	0/1, 2		0.73 (0.24–2.21)	0.583		
Tumor locarion (head/body or tail)	Head/body or tai		1.46 (0.62–3.42)	0.389		
CEA after chemotherapy						
	<5.0/5.0 ≤ ng/ml		0.18 (0.06–0.52)	0.002*	0.14 (0.04–0.48)	0.002*
CA19‐9 after chemotherapy						
	< 100/100 ≤ U/ml		0.18 (0.06–0.53)	0.021*	0.25 (0.06–0.96)	0.043*
Operation	PD, TP/DP		1.46 (0.62–3.42)	0.389		

Abbreviations: ASA‐PS, American Society of Anesthesiologists physical status; BMI, body mass index; CR, complete response; DP, distal pancreatectomy; mGPS, modified Glasgow Prognostic Score; NLR, neutrophil‐to‐lymphocyte ratio; PD, pancreatoduodenectomy; PNI, prognostic nutritional index; PR, partial response; SD, stable disease; TP, total pancreatectomy.

* *p* values are statistically significant.

### 3.4 Recurrence Pattern After Operation With Preoperative Chemotherapy

Recurrence was found in 21 cases (75.0%), with a median disease‐free survival (DFS) of 8.1 months (range: 2.3–95.4 months). Of the 21 cases with recurrence, most of the cases (*n* = 19, 90.5%) showed recurrence within 24 months after resection; however, two cases showed recurrence 54.7 and 84.4 months after resection. The most frequent sites of recurrence site were the liver (*n* = 12, 57.1%), lung (*n* = 5, 23.8%), peritoneum (*n* = 4, 19.0%), local (*n* = 3, 14.3%), lymph nodes (*n* = 3, 14.3%), and bone (*n* = 1, 4.8%). In addition, among patients who achieved complete response (CR) for liver oligometastases, six cases showed recurrence. The most common site of recurrence was the liver (three cases), followed by the peritoneum (two cases), the lung (two cases), and local recurrence (one case). The frequency of liver recurrence was not significantly different between patients who achieved CR for liver oligometastases and those who did not (*p* = 0.903).

## 4 Discussion

Although liver oligometastasis has been the focus of increasing attention, there is no data on liver oligometastasis alone; it has instead been reported as a part of the broader topic of liver metastasis. This is the largest collection of data on resected liver oligometastases from a national cohort study. We aimed to evaluate the efficacy of resecting liver oligometastatic pancreatic cancer. We found that several patients who met the specific criteria, such as low CA19‐9, low CEA, and resectability within the BR‐PV, benefited from surgical treatment after chemotherapy.

Current guidelines described that distant metastasis, regardless of resectability, recommend systematic chemotherapy as the standard treatment [6]. Oligometastatic disease has been proposed as a distinct cancer state between locally confined and systemically metastasized disease [10, 11]; however, its biology is not well understood. The role of preoperative chemotherapy in improving the survival prognosis of pancreatic cancer has evolved in BR pancreatic cancer [14, 15, 16, 17]; however, there have been no reports focusing solely on liver oligometastatic pancreatic cancer. Therefore, we evaluated the survival benefit of preoperative chemotherapy in these cases and found that patients who received preoperative chemotherapy had a significantly better prognosis than those who underwent upfront surgery, suggesting that preoperative chemotherapy is essential for liver oligometastatic pancreatic cancer. We found that the median OS was 37.2 months, and the univariate analysis showed that CA19‐9 (< 100 U/mL), low CEA (< 5 U/mL), and resectability within the BR‐PV were associated with a significantly better prognosis. If any of these criteria were met, the median OS was > 50 months. Furthermore, the multivariate analysis revealed that these three factors were independent positive prognostic factors. Takeda et al. reported prognostic factors for OS in patients with liver oligometastases and identified age < 70 years, Eastern Cooperative Oncology Group (ECOG) performance status (PS) of 0, modified Glasgow prognostic score (mGPS) of 0, and CA19‐9 < 1000 U/mL as prognostic factors. Although these factors differed from our results, most patients in their cohort did not undergo surgical treatment, and they insisted that the factors stratified the prognosis of patients with liver oligometastasis. In contrast, all patients in our cohort underwent surgical resection; therefore, their age and nutritional status, such as the mGPS and ECOG PS, may have already met these criteria. Takeda et al. [9] reported 10 cases of resected liver oligometastases, with a median OS of 54.6 months, which is comparable to our results. This suggests that some patients were long‐term survivors. In that report, CA19‐9 normalization was observed in all patients before surgery. Therefore, a reduction in CA19‐9 levels after chemotherapy may be a key marker for selecting patients for surgery.

Regarding the response of liver oligometastases to chemotherapy, although not a series of oligometastases, Takeda et al. [9] reported that 12 of 13 (92%) patients had no liver metastasis. Satoi et al. [7] reported that the presence of liver metastasis was post‐operatively confirmed in 1 of 10 patients with surgical resection after systemic chemotherapy for 7 months or longer. Furthermore, Hank et al. [18] reported that 48% of liver metastases had disappeared (ypM0) and that ypM0 was an independent prognostic factor. In this study, we found CR of liver oligometastasis in eight patients (28%), and pathological CR for liver metastasis was not an independent factor for prognosis. Confirming liver metastasis would be difficult with imaging diagnosis alone, and there may be false positives, such as cholangitis. In our cohort, most patients did not undergo pathological confirmation at the initial diagnosis, and the ratio of pathological confirmation before surgery was unknown in the previous series. Ideally, preoperative confirmation with needle biopsy is preferable; however, it is difficult to perform this procedure in all patients. The issue of false negatives also exists. Considering these situations, a multimodal imaging diagnosis is important, and if liver oligometastasis is suspected, preoperative chemotherapy is recommended.

Regarding the duration of preoperative chemotherapy, no significant difference in prognosis was observed, regardless of whether the threshold was 3 or 6 months. To date, most reports on surgical treatment have documented > 6 months of preoperative chemotherapy [7, 9]. In our cohort, although half of the patients received chemotherapy for > 6 months, the other half received it within 6 months, with a median OS of 40.5 months (9.9–105.3 months). In addition, eight patients received preoperative chemotherapy for < 3 months, and four patients survived for > 50 months. Satoi et al. [7] reported five cases of oligo and occult liver metastases treated with short‐term neoadjuvant chemotherapy (< 3 months). Although the median OS was only 12 months, one patient survived for 85.5 months with adjuvant chemotherapy. All four patients had CA19‐9 levels within 100 U/mL and CEA levels within 5.0 U/mL. Considering these findings, surgical treatment might be acceptable even if preoperative chemotherapy is administered within 6 months, provided that the criteria in this study are met.

In this study, we did not evaluate the benefits of adjuvant chemotherapy because most of the patients (85.7%) received adjuvant chemotherapy, making it difficult to evaluate its efficacy. In addition, evaluating the duration of adjuvant chemotherapy is challenging as it is usually intended to last for at least 6 months. A shorter duration will indicate that the regimen had changed owing to recurrence. Therefore, it is not surprising that patients who received shorter adjuvant chemotherapy durations had shorter prognoses than those who underwent longer adjuvant chemotherapy durations.

In this study, we found that surgical treatment for liver oligometastatic pancreatic cancer was effective, and the prognosis was favorable if certain conditions were met. However, the recurrence rate was high, which is similar to that in previous reports [9]. Even if long‐term survival is expected, once recurrence occurs, most patients cannot survive. In Japan, most patients receive S‐1 as adjuvant chemotherapy; therefore, other regimens are needed to prevent recurrence.

This study had some limitations. First, this is the retrospective study, and only resected cases were included. Patients with oligometastasis were excluded due to progression of the metastasis. Second, although it was a national cohort and the largest case series of liver oligometastasis, it included a limited number of patients. A larger‐scale analysis and a prospective comparison with patients undergoing non‐surgical treatment are necessary to validate these findings. Finally, occult liver metastases, which were not detected radiologically and were found at the time of surgery, were included in this study. Hashimoto et al. [19] reported that the prognosis of patients with occult liver metastases was better than that of patients with radiologically defined liver metastases. Future studies should differentiate between these two types of metastases.

In summary, this is the largest study to date to analyze only liver oligometastases treated with resection. Preoperative chemotherapy is essential for patients with oligometastatic pancreatic cancer. Despite high recurrence rates, patients who meet the specific criteria are expected to have a favorable prognosis.

## Conflicts of Interest

The authors declare no conflicts of interest.

## Data Availability

The authors have nothing to report.
